# The triglyceride and glucose index (TyG) is an effective biomarker to identify nonalcoholic fatty liver disease

**DOI:** 10.1186/s12944-017-0409-6

**Published:** 2017-01-19

**Authors:** Shujun Zhang, Tingting Du, Jianhua Zhang, Huiming Lu, Xuan Lin, Junhui Xie, Yan Yang, Xuefeng Yu

**Affiliations:** 1 Division of Endocrinology, Department of Internal Medicine, Tongji Hospital, Tongji Medical College, Huazhong University of Science and Technology, 1095 Jiefang Avenue, Wuhan, 430030 China; 2 Department of Health Examination, Wuhan Iron and Steel Company (WISCO) General Hospital, 29 Metallurgical Avenue, Wuhan, 430080 China; 3 Department of Endocrinology, Wuhan Iron and Steel Company (WISCO) General Hospital, 29 Metallurgical Avenue, Wuhan, 430080 China

**Keywords:** The triglyceride and glucose index, Nonalcoholic fatty liver disease, Alanine aminotransferase, Insulin resistance

## Abstract

**Background:**

The triglyceride and glucose index (TyG) has been proposed as a marker of insulin resistance. We aimed to investigate the ability of TyG, through comparing with the predictive value of alanine aminotransferase (ALT), to identify individuals at risk for nonalcoholic fatty liver disease (NAFLD).

**Methods:**

A cross-sectional study was conducted in a Chinese health examination cohort of 10 761 people aged above 20 years. NAFLD was diagnosed by ultrasonography.

**Results:**

Compared with the participants in the lowest quartile of TyG, the adjusted odds ratios (ORs) and 95% confidence intervals (CIs) for NAFLD were 1.8 (1.5–2.1), 3.0 (2.5–3.5), and 6.3 (5.3–7.5) for those in the second, the third, and the fourth quartile of TyG, whereas the corresponding ORs (95% CI) for NAFLD were 1.5 (1.3–1.7), 1.9 (1.6–2.2), and 3.1 (2.6–3.7) for the upper three quartiles of ALT. These results suggested that TyG was superior to ALT in association with NAFLD risk. According to the ROC analysis, the optimal cut-off point of TyG for NAFLD was 8.5 and the area under the ROC curve (AUC) was 0.782 (95% CI 0.773–0.790), with 72.2 and 70.5% sensitivity and specificity, respectively. The AUC of TyG was larger than that of ALT (0.715 (95% CI 0.705–0.725), *P* for difference <0.0001), whereas the largest AUC was obtained when adding TyG to ALT (0.804 (95% CI 0.795–0.812), *P* for difference <0.0001).

**Conclusions:**

TyG is effective to identify individuals at risk for NAFLD. A TyG threshold of 8.5 was highly sensitive for detecting NAFLD subjects and may be suitable as a diagnostic criterion for NAFLD in Chinese adults.

**Electronic supplementary material:**

The online version of this article (doi:10.1186/s12944-017-0409-6) contains supplementary material, which is available to authorized users.

## Background

With the rapidly economic growth and lifestyle changing, nonalcoholic fatty liver disease (NAFLD) is becoming a major health hazard over the world. It encompasses a spectrum of hepatic pathologies ranging from simple hepatic steatosis to steatohepatitis and cirrhosis due to non-alcoholic causes, with increased levels of hepatic enzymes such as alanine aminotransferase (ALT), aspartate aminotransferase (AST), or γ-glutamyltransferase (GGT) [1]. NAFLD is closely associated with obesity, dyslipidaemia and type 2 diabetes mellitus [1, 2], and simultaneously forecasts the uptrend of cardiovascular disease risk [3]. Thus, early identification for the risk of NAFLD is important for public health and a simple and effective diagnostic tool would be useful to early detect and manage the NAFLD subjects.

NAFLD is considered as the liver manifestation of metabolic syndrome and the two key components of metabolic syndrome, triglycerides (TG) and fasting plasma glucose (FPG) are overproduced by the fatty liver [4]. Recently, the triglyceride and glucose index (TyG), also taken as the product of TG and FPG, has been recommended as a reliable and simple surrogate index for insulin resistance [5, 6]. The association between the TyG index and liver steatosis has been demonstrated in a cohort of 50 asymptomatic women from Mexico [7], however, whether the TyG index is able to detect NAFLD risk in Chinese adults has not been investigated.

Among biochemical measurements, serum ALT appears to be one liver enzyme that is most relevant to liver fat content [8], and it is used as a simple marker for hepatic steatosis in large-scale screening studies [9–11]. Therefore, we aimed to characterize and compare the usefulness of TyG and ALT for identifying individuals with the risk of NAFLD in a large cohort of Chinese adults who underwent health examination.

## Methods

### Subjects and study design

The study participants were Chinese employees aged above 20 years from Wuhan Iron and Steel Company (WISCO). As our previous studies introduced [12, 13], the data came from health examination of all employees and retired workers at the WISCO General Hospital in 2009. Questionnaires were utilized for demographic characteristics such as age, sex, medical history, family history, and smoking and drinking status. The exclusion criteria included carrying hepatic virus infections, autoimmune hepatic disease, other chronic hepatic diseases, taking medicines for established diabetes and dyslipidaemia, missing data on age, sex, blood pressure (BP), body mass index (BMI), FPG, TG, ALT or B-ultrasonic examination for liver. Finally, there were 10,761 participants included in present analysis, consisting of 6,758 men and 4,003 women. The fact that 62.8% of the participants were men was in consistent with the proportion of male employees at WISCO. Our study was approved by institutional review board of WISCO General Hospital, and the informed consent requirement was exempted because of our retrospective estimation of de-identified database.

### Clinical measurements

As our previous studies described, physical examination was performed and anthropometry was obtained comprised of weight, height, and BP. BMI was calculated as weight (in kilograms)/height square (in metres). Fasting blood samples were collected after at least 10 h overnight and analyzed for the biochemical measurements, such as ALT, FPG, uric acid (UA), white blood cell (WBC) count, hepatitis viral antigen/antibody, and serum lipids, including TG, total cholesterol (TC), high-density lipoprotein cholesterol (HDL-C), and low-density lipoprotein cholesterol (LDL-C). All the measurements were determined by an auto-analyzer (Hitachi 7600, Ltd, Tokyo, Japan). The product of triglyceride and glucose was calculated as established formulas, TyG = Ln [TG (mg/dl) FPG (mg/dl)/2] [5].

### Definitions for NAFLD and metabolic disorders

According to guidelines proposed by the Asia-Pacific Working Party [14], NAFLD was diagnosed by the presence of fatty liver, and simultaneously ruled out excessive alcohol intake (>140 g/week for men, >70 g/week for women) and the history of carrying hepatic virus and utilization of steatogenic or hepatotoxicity medicines. Fatty liver was assessed as the presence or absence of hepatic steatosis by ultrasound scan, identified by one professional operator using standard method, the presence of increased echogenicity of liver compared to renal cortex.

BMI was classified into two groups according to the World Health Organization (WHO) Asia-Pacific guidelines [15]: non-obese (normal weight/overweight) (BMI < 25 kg/m^2^) and obese (BMI ≥ 25 kg/m^2^). Hypertension was defined as systolic/diastolic BP ≥140/90 mmHg or use of antihypertensive drugs [16]. Hyperuricaemia was defined as serum uric acid over 420 μmol/L for male, and 360 μmol/L for female [17]. High WBC count was considered as the highest quartile of WBC count in the present study (the highest quartile: >6.8 × 10^9^/L).

### Statistical analysis

All statistical analyses were performed by SPSS version 20.0 (Chicago, IL, USA). Normality testing was conducted, and continuous variables were expressed as median and interquartile ranges (IQR) because of their skew distribution, while categorical variables were presented as percentages. Differences between NAFLD participants and non-NAFLD individuals were assessed using Mann–Whitney *U* test for continuous variables and chi-square test for categorical variables.

Binary logistic regression analysis was conducted to calculate odds ratio (OR) and 95% confidence intervals (CI) for NAFLD in different TyG and ALT quartiles. Four models were applied: model 1 was unadjusted. Model 2 was adjusted for age, sex and BMI. Model 3 was adjusted for all variables in model 2 plus systolic BP, UA and WBC. Model 4 was adjusted for all variables in model 3 plus TyG for ALT quartiles or plus ALT for TyG quartiles. The multi-variable adjusted ORs and corresponding 95% CIs for NAFLD associated with the highest quartile of TyG or ALT, compared with the lower three quartiles, were further estimated in subgroups classified by sex, age, BMI, BP, UA, and WBC.

Finally, we performed the receiver operating characteristic (ROC) curve analysis to test the ability of TyG to diagnose NAFLD. The sensitivity, specificity, and Youden index of TyG were calculated, and the optimal cut-off value of TyG for detecting NAFLD was derived from the point with the maximum Youden index. Comparisons between the areas under the ROC curve (AUC) of TyG and ALT, as well as TyG plus ALT were conducted by the method described by DeLong et al [18].

A 2-tailed *P* value <0.05 was considered significant.

## Results

### Characteristics of the study population

In this population, the mean age was 49.5 (±14.9) years and mean BMI 23.7 (±3.1) kg/m^2^. There were 4,349 participants diagnosed as NAFLD by liver ultrasonic examination, with a prevalence of 40.4%. Clinical characteristics of the study participants according to NAFLD category were described in Table 1. Compared to non-NAFLD individuals, NAFLD persons were more likely to be older, and to have a higher proportion of men, as well as to have an adverse cardiometabolic risk profile, such as higher BMI, BP, FPG, UA, TG, TC and LDL-C, and lower HDL-C (all *P* < 0.0001). Notably, the median values of TyG index and ALT were both significantly elevated in subjects with NAFLD in contrast to those without the disease (both *P* < 0.0001).

**Table 1:** Characteristics of the participants according to presence of NAFLD

	Non-NAFLD	NAFLD	*P* value
Total, *N* (%)	6 412 (59.6%)	4 349 (40.4%)	—
Men, *N* (%)	3 622 (56.5%)	3 136 (72.1%)	<0.0001
Age, years	47.0 (36.0–57.0)	52.0 (43.0–59.0)	<0.0001
Body mass index, kg/m^2^	22.1 (20.4–23.8)	25.6 (24.0–27.4)	<0.0001
Systolic blood pressure, mmHg	120.0 (110.0–130.0)	128.0 (120.0–139.0)	<0.0001
Diastolic blood pressure, mmHg	75.0 (70.0–80.0)	80.0 (75.0–90.0)	<0.0001
Fasting plasma glucose, mmol/L	4.9 (4.6–5.3)	5.2 (4.8–5.8)	<0.0001
Triglycerides, mmol/L	0.9 (0.7–1.3)	1.6 (1.1–2.4)	<0.0001
Total cholesterol, mmol/L	4.4 (3.8–5.0)	4.8 (4.2–5.4)	<0.0001
HDL cholesterol, mmol/L	1.4 (1.2–1.6)	1.2 (1.1–1.4)	<0.0001
LDL cholesterol, mmol/L	2.6 (2.2–3.1)	3.0 (2.5–3.5)	<0.0001
Uric acid, μmol/L	282.0 (233.0–334.0)	333.0 (284.9–384.6)	<0.0001
White blood cell counts, ×10^9^/L	5.5 (4.7–6.5)	6.1 (5.3–7.2)	<0.0001
Alanine aminotransferase, U/L	17.0 (13.0–24.0)	26.0 (18.0–37.0)	<0.0001
TyG	8.2 (7.8–8.6)	8.8 (8.4–9.3)	<0.0001

Data are presented as median (interquartile range) or percentage

TyG indicates the triglycerides and glucose index

*NAFLD*: nonalcoholic fatty liver disease; *HDL*; high density lipoprotein; *LDL*; low density lipoprotein

### Association between the TyG index and NAFLD risk

**Fig. 1:**
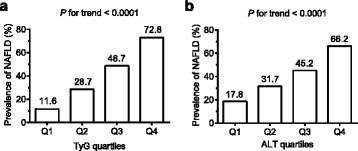
Prevalence of NAFLD according to the quartiles of TyG (**a**) or ALT (**b**). Classification of TyG quartiles: Q1 (~8.0), Q2 (8.1 ~ 8.4), Q3 (8.5 ~ 8.9), Q4 (9.0~); ALT (U/L) quartiles: Q1 (~14.0), Q2 (14.1 ~ 20.0), Q3 (20.1 ~ 29.0), Q4 (29.1~). NAFLD, nonalcoholic fatty liver disease; ALT, alanine aminotransferase; Q1, first quartile; Q2, second quartile; Q3, third quartile; Q4, fourth quartile

The prevalence of NAFLD was significantly increased along with the increasing levels of TyG. The prevalence in individuals of the highest TyG quartile was 72.8%, which showed a 6.3-fold increase as compared with that of the ones in the lowest quartile (Fig. 1a). We also observed a significant trend of increasing odds ratio for NAFLD with increasing levels of TyG in Table 2. The crude ORs for NAFLD (model 1) were 3.0 (2.6–3.5), 7.2 (6.3–8.3), and 20.4 (17.6–23.6) among subjects in the second, the third, and the fourth quartile of TyG, as compared to persons in the first TyG quartile (*P* for trend <0.0001). The ORs were dramatically decreased but the results remained significant after adjusted for age, sex and BMI (model 2). The associations persisted, even though they were slightly attenuated, after additional adjustment for systolic BP, UA and WBC (model 3), and further adjustment for ALT (model 4).

**Table 2:** Odds ratios for NAFLD in different quartiles of TyG index or ALT

		Model1	Model2	Model3	Model4
TyG	Q1 (Reference)	1	1	1	1
	Q2 (OR, 95% CI)	3.0 (2.6–3.5)	1.9 (1.6–2.2)	1.8 (1.5–2.1)	1.8 (1.5–2.1)
	Q3 (OR, 95% CI)	7.2 (6.3–8.3)	3.4 (2.9–4.1)	3.1 (2.6–3.6)	3.0 (2.5–3.5)
	Q4 (OR, 95% CI)	20.4 (17.6–23.6)	8.1 (6.8–9.6)	6.8 (5.7–8.1)	6.3 (5.3–7.5)
ALT	Q1 (Reference)	1	1	1	1
	Q2 (OR, 95% CI)	2.0 (1.8–2.3)	1.6 (1.3–1.8)	1.6 (1.3–1.8)	1.5 (1.3–1.7)
	Q3 (OR, 95% CI)	3.6 (3.2–4.1)	2.1 (1.8–2.5)	2.1 (1.8–2.4)	1.9 (1.6–2.2)
	Q4 (OR, 95% CI)	8.6 (7.6–9.7)	4.1 (3.5–4.8)	3.8 (3.2–4.4)	3.1 (2.6–3.7)

Model 1 was unadjusted

Model 2 was adjusted for age, sex and body mass index

Model 3 was adjusted for all variables in model 2 plus systolic blood pressure, uric acid and white blood cell counts

Model 4 was adjusted for all variables in model 3 plus TyG for ALT quartiles or plus ALT for TyG quartiles

*NAFLD*: nonalcoholic fatty liver disease; *ALT*: alanine aminotransferase; *Q*1: first quartile; *Q*2: second quartile; *Q*3: third quartile; *Q*4: fourth quartile

**Fig. 2:**
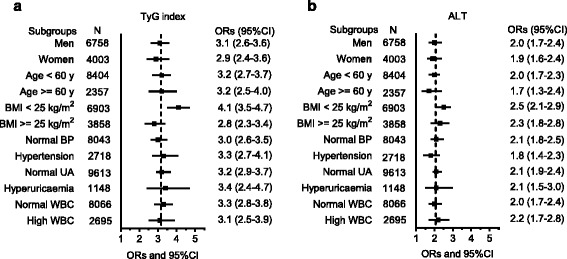
Odds ratios for NAFLD associated with TyG (**a**) or ALT (**b**) in various subgroups. The odds ratios were adjusted for potential risks of interest except for stratified variable in each subgroup. NAFLD, nonalcoholic fatty liver disease; ALT, alanine aminotransferase; BMI, body mass index; BP, blood pressure; UA, uric acid; WBC, white blood cells

We also assessed the relationship between TyG of the top quartile, as compared with the three lower quartiles, and odds of NAFLD in diverse subgroups (Fig. 2a). When controlling for potential risk factors, the greater ORs of TyG in the highest quartile for NAFLD were consistently seen in every evaluated subgroup, especially in subgroup of BMI <25 kg/m^2^. Meanwhile, the greater ORs were comparable between men and women, as well as between different age-, BP-, UA-, and WBC-subgroups.

### Comparison of the associations of TyG and ALT with NAFLD risk

As expected, the prevalence of NAFLD was significantly increased with the increasing levels of ALT (Fig. 1b). The prevalence in people of the fourth quartile of ALT was 66.2%, which rose to 3.7 times as compared with that of ones in the lowest ALT quartile. Similar to TyG, a trend toward higher risk of NAFLD was observed as ALT increased. However, the adjusted ORs for NAFLD of the second to fourth TyG quartiles (1.8 (1.5–2.1), 3.0 (2.5–3.5), and 6.3 (5.3–7.5), respectively) were greater than corresponding figures of ALT quartiles (1.5 (1.3–1.7), 1.9 (1.6–2.2), and 3.1 (2.6–3.7), respectively) (Table 2). Furthermore, in the subgroup analysis, the greater ORs for NAFLD of ALT in the highest quartile were consistently seen in every evaluated subgroup, although these ORs for NAFLD were less than those of the corresponding figures of TyG (Fig. 2b).

When the subjects were divided into 16 groups by TyG and ALT quartiles, combined effect of TyG and ALT in identifying individuals with NAFLD risk was seen (Table 3). The multivariable-adjusted ORs for NAFLD significantly increased along with increasing quartiles of both ALT and TyG (*P* for interaction <0.05). Individuals in the highest quartile of ALT by the highest quartile of TyG had a much higher risk of NAFLD (OR 17.3 (95% CI 12.2–24.4)) than those in the lowest ALT quartile by the lowest TyG quartile. Importantly, the ORs for NAFLD increased more quickly across ordinal TyG quartiles in each ALT quartile, as compared with the ones across ALT quartiles in each TyG quartile.

**Table 3:** Adjusted odds ratios for NAFLD in four TyG quartiles by ALT quartiles

		TyG			
		Q1 (OR, 95% CI)	Q2 (OR, 95% CI)	Q3 (OR, 95% CI)	Q4 (OR, 95% CI)
ALT	Q1 (OR, 95% CI)	1	1.4 (1.0–2.0)	2.2 (1.5–3.2)	5.0 (3.4–7.2)
	Q2 (OR, 95% CI)	1.2 (0.8–1.8)	2.3 (1.6–3.3)	3.5 (2.5–4.9)	7.4 (5.2–10.7)
	Q3 (OR, 95% CI)	1.5 (1.0–2.2)	2.9 (2.1–4.0)	4.6 (3.3–6.4)	8.9 (6.3–12.4)
	Q4 (OR, 95% CI)	2.2 (1.5–3.4)	4.1 (2.9–5.9)	7.9 (5.6–11.1)	17.3 (12.2–24.4)

Adjusted variables included age, sex, body mass index, systolic blood pressure, uric acid and white blood cell counts

*NAFLD*: nonalcoholic fatty liver disease; *AL*: alanine aminotransferase; *Q*1: first quartile; *Q*2: second quartile; *Q*3: third quartile; *Q*4: fourth quartile

### Diagnostic accuracy of ALT and TyG for NAFLD

Using elevated ALT (≥40.0 U/L, the upper limit of reference range) as the diagnostic tool for NAFLD in this population yielded a prevalence of 12.5% (1 348 cases/10 761). Compared with ultrasonography, the sensitivity of ALT was 22.0% (957/4 349 cases) for detecting individuals with ultrasound-diagnosed NAFLD, with a specificity of 93.9%. Using the ROC curve, the optimal cut-off point of ALT was 20.5 U/L, yielding sensitivity and specificity of 66.1 and 65.9%, respectively. The AUC of ALT for predicting NAFLD was 0.715 (95% CI 0.705–0.725) (*P* < 0.0001).

**Fig. 3:**
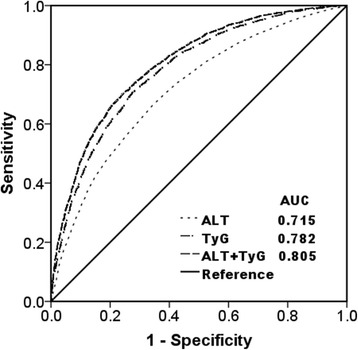
Receiver operative characteristic (ROC) curves and corresponding areas under the curve (AUC) for NAFLD. The AUC of TyG, ALT, and TyG plus ALT were 0.782 (95% CI 0.773–0.790), 0.715 (95% CI 0.705–0.725), and 0.804 (95% CI 0.795–0.812), respectively. *P* values for the difference of any two AUCs were <0.0001. NAFLD, nonalcoholic fatty liver disease; ALT, alanine aminotransferase

In contrast, the AUC of TyG was significantly higher than that of ALT (0.782 (95% CI 0.773–0.790); *P* < 0.0001 for the difference of the AUCs). The optimal cut-off point of TyG was 8.5, with a sensitivity of 72.2% and specificity of 70.5%. Additionally, when TyG was added to ALT (TyG + ALT model), the AUC was higher than that of TyG and ALT alone (0.804 (95% CI 0.795–0.812), *P* < 0.0001 for the difference of the AUCs), with 71.0 and 74.9% sensitivity and specificity (Fig. 3).

### Subgroup analysis according to ALT

Elevated ALT is common in nonalcoholic steatohepatitis, thus, we further divided the population into two subgroups (ALT <40 U/L as normal ALT group and >40 U/L as elevated ALT group) and compared the predictive efficiency of TyG and ALT, respectively. Patients with NAFLD had significantly higher levels of TyG and ALT than non-NAFLD subjects in both normal (TyG, 8.8 vs. 8.2; ALT, 22.0 vs. 16.0 U/L; both *P* < 0.0001) and elevated (TyG, 9.0 vs. 8.3; ALT, 53.0 vs. 49.0 U/L; both *P* < 0.0001) ALT groups. The fourth quartile of TyG was associated more closely with NAFLD risk than the fourth quartile of ALT in either normal (adjusted ORs for TyG vs. ALT: 5.6 (4.7–6.8) vs. 2.4 (2.0–2.9)) or elevated ALT group (adjusted ORs: 7.3 (4.3–12.3) vs. 1.2 (0.7–1.8)) (Additional file 1: Table S1). The ROC analysis also showed that TyG was superior to ALT in predicting NAFLD in both subgroups. Notably, the diagnostic value of ALT was markedly weaken in elevated ALT group (AUC = 0.562) than in normal ALT group (AUC = 0.684), whereas the predictive ability of TyG was slightly strengthened in elevated ALT group (AUC = 0.787 vs. 0.769 for normal ALT group) (Additional file 1: Table S2).

## Discussion

In the present study, we observed a strong and positive association between TyG and risk of NAFLD, after adjustment for potential confounders. We also demonstrated that TyG could detect NAFLD accurately with a AUC of 0.782 (0.773–0.790) and the optimal cut-off point of TyG for diagnosing NAFLD was 8.5, with a sensitivity of 72.2% and specificity of 70.5% in Chinese. The TyG index was much superior to ALT for identifying NAFLD in the population. Furthermore, adding TyG to ALT had an even better performance to detect subjects at risk for NAFLD. Thus, TyG could be an effective noninvasive method for the identification of NAFLD.

In our study, ALT was associated with NAFLD risk as anticipated. Except for the intimate correlation with liver fat content, ALT not only has high specificity for liver injury, but also is considered to be a nontraditional cardiometabolic risk factor, associated with type 2 diabetes, metabolic syndrome and subsequent cardiovascular disease [19, 20]. In recent guidelines recommended by EASL-EASD-EASO [21], liver enzymes, except for ultrasound, is suggested to be part of routine work-up for screening NAFLD in subjects with obesity or metabolic syndrome. Moreover, Recent studies reported that ALT was associated with markers of oxidative stress and inflammation which can lead to insulin resistance and further promote excessive accumulation of triglycerides in liver [22, 23]. However, there were studies suggesting that ALT is not a sensitive marker for NAFLD. One study suggested that 79% of subjects with hepatic steatosis had normal levels of ALT [24] and the sensitivity of ALT for the diagnosis of primary NAFLD is much lower, as compared with ultrasonography [25]. In accordance with prior studies, our findings showed that only 22.0% of individuals with NAFLD had increased levels of ALT (≥40 U/L). This result implicated that elevated ALT is probably inadequate to identify the individuals with NAFLD. It is, therefore, important to find more sensitive biomarker other than ALT for detect individuals with NAFLD in clinical practice.

Our data suggested that TyG performed better than ALT to discriminate NAFLD as indicated by the present study that the TyG had a stronger association with risk of NAFLD and a larger AUC for diagnosing NAFLD, as compared with ALT. This observation is not surprising since the TyG index, derived from TG and FPG, takes into consideration the two crucial metabolic variables altered in fatty liver, and highly correlates with insulin resistance [6], the key pathogenesis of NAFLD. Recently, there is growing interest in the TyG index. Several studies have reported that the TyG index was associated with the development of diabetes and cardiovascular diseases [26, 27]. Although the TyG index mathematical model was developed in Mexican population, our prior study showed that the TyG index was effective to identify the risk of insulin resistance (assessed by homeostasis model assessment of insulin resistance) in Chinese individuals [28]. Importantly, in the SAM study, Gastaldelli et al. [29] proposed the TyG was not a good measure of peripheral insulin resistance but rather of hepatic insulin resistance since it was well correlated with the amount of hepatic fat. The present study observed that high level of TyG was associated with increased risk of NAFLD in a dose-response manner. Actually, prior studies have shown that TyG was independently associated with hepatic steatosis in chronic hepatitis C patients and NAFLD patients [30, 31], which is agreeable with our present study. More recent study conducted by Simental-Mendia et al. [7] also suggested that TyG is effective to screen simple steatosis in 50 asymptomatic women and is superior to other indices for NAFLD, including SteatoTest, NashTest, and Fatty liver index. However, the small size of the cohort and lack of men participants limited the generalization of the results. Thus, further validating the ability of TyG in identifying NAFLD in large and diverse population is needed. In the present study, we determined in a large Chinese population that a threshold of TyG ≥8.5 was effective enough to identify NAFLD individuals, with an AUC of 0.782. Notably, the cut-off point of 8.5 was quite different from the finding of 4.58 by Simental-Mendia [7], the reason for this may be explained by the different metabolic status among subjects from the two studies or the different ethnicities. The TyG level in our study was similar to that of other population from Korea [27, 32], America [33], France [30], and Spain [26], whereas the level of TyG in studies from Mexico was close to that of participants from Italy [31]. We also found that the diagnostic accuracy of TyG was superior to ALT, even if the optimal cut-off point of ALT was 20.5 U/L with a sensitivity of 66.1% and specificity of 65.9%. NAFLD is closely associated with obesity and metabolic syndrome and characterized by excessive accumulation of triglycerides in the liver, which leads to hepatic insulin resistance. In turn, the condition of insulin resistance in liver leads to overproduction of fasting plasma glucose and very low density lipoprotein (VLDL), which contains rich triglycerides present in serum [4]. Our results, actually, showed that NAFLD individuals had significantly higher BMI, FPG and triglycerides levels than those with non-NAFLD. Based on above observations, it is reasonable to use TyG index, a product of triglycerides and FPG, as an effective diagnostic tool for NAFLD.

The TyG index was also superior to ALT in identifying NAFLD in individuals with elevated ALT (≥40 U/L). Serum level of ALT is commonly used as a surrogate indicator for evaluating liver histology in various liver diseases, and ALT seems to be more closely associated with steatohepatitis than other biomarkers, e.g. cytokeratin-18 [34]. That is, in elevated ALT group, NAFLD subjects are suspected as suffering from steatohepatitis. Therefore, our results indicated that TyG was an efficacy predictor for NAFLD (including both simple steatosis and steatohepatitis).

The predicting efficacy of TyG for risk of NAFLD was partially affected by BMI of the individuals. As we showed in Table 2, ORs in model 1 were dramatically decreased when they were adjusted for age, sex and BMI in model 2. However, subgroup analysis indicated that the association of TyG and NAFLD risk was significantly stronger in non-obese subjects than that in obese ones, but this was not the case in subgroups with different sex and age. These results, therefore, suggested that BMI is an important factor affected TyG efficacy for identifying the individuals with risk of NAFLD.

The results of the present study also showed that TyG could improve diagnostic accuracy of ALT for detecting NAFLD as evidenced by enlarged AUC when adding TyG to ALT. This finding further verified the effectiveness of TyG in the diagnosis of NAFLD. Simultaneously, it was likely to have a synergy effect between TyG and ALT in association with NAFLD, as indicated by the significant interaction between TyG and ALT on the risk of NAFLD in Table 3. Moreover, our findings of the model 4 in Table 2 showed that ALT and TyG index were two independent variables for predicting the risk of NAFLD, notwithstanding, these two markers may influence each other and contribute to the same pathogenic mechanism leading to the development and progression of NAFLD.

The strength of our study includes the relative large sample size, the diverse subgroups analysis, and the comprehensive analysis of the variables. There are several limitations in the present study. First, we recognized NAFLD only by ultrasonography, which has limited sensitivity and could not reliably detect steatosis when liver fat infiltration <20% [35, 36] or in obese individuals especially with BMI > 40 kg/m^2^ [37]. However, in clinical practice, ultrasound is proposed as a preferable imaging method for screening NAFLD individuals, as it is cheaper and more widely available [21]. Second, the study was cross sectional so that a causal relationship cannot be obtained and the participants in the present study came from selected populations (industrial employees and retired workers) with a preponderance of men; therefore, extrapolating the findings to the Chinese general population or to other races or ethnicities should be interpreted cautiously. Third, the information about abdominal obesity, i.e. waist circumference, was not available, therefore, we could not compare the efficacy between TyG and other clinical indexes, such as fatty liver index and lipid accumulation products, which is an important issue and further studies are needed. Finally, the ability of TyG in detecting steatohepatitis and advanced fibrosis was not able to be evaluated in the present study due to the lack of liver biopsy. However, Simental-Mendia et al. [7] found that TyG was a better screening method for simple steatosis and steatohepatitis, as compared to other well-known clinical markers for NAFLD. At the same time, due to the limited sample size of that study, it is necessary to further verify in large and various population.

## Conclusions

We validated the effectiveness of the TyG index in identifying individuals at risk for NAFLD in a large sample of Chinese participants. We determined that the optimal cut-off point of TyG for identifying NAFLD was 8.5 among Chinese adults. Our findings have important clinical implications. Liver biopsy was invasive and the imaging examination for NAFLD is not practical and convenient in epidemiologic studies and some retrospective cohorts, and serum biomarkers are preferred for large scale screening studies, hence the TyG index may be widely used for identification and subsequent management of individuals with NAFLD.

## Additional file

Additional file 1: Table S1. Odds ratios for NAFLD in different quartiles of TyG index or ALT in ALT <40 and ≥40 U/L groups. Table S2 Diagnostic value of TyG and ALT for NAFLD in ALT <40 and ≥40 U/L groups. (DOCX 19 kb)

## Abbreviations

ALT: Alanine aminotransferase
AST: Aspartate aminotransferase
AUC: Areas under the curve
BMI: Body mass index
BP: Blood pressure
CI: Confidence intervals
FPG: Fasting plasma glucose
GGT: γ-glutamyltransferase
HDL-C: High-density lipoprotein cholesterol
IQR: Interquartile ranges
LDL-C: Low-density lipoprotein cholesterol
NAFLD: Nonalcoholic fatty liver disease
OR: Odds ratio
ROC: Receiver operating characteristic
TC: Total cholesterol
TG: Triglycerides
UA: Uric acid
VLDL: Very low density lipoprotein
WBC: White blood cell
