# Supplementary material for: The triglyceride and glucose index (TyG) is an effective biomarker to identify nonalcoholic fatty liver disease
Source: Lipids Health Dis. 2017 Jan 19;16:15. doi: 10.1186/s12944-017-0409-6 (PMC5248473; doi:10.1186/s12944-017-0409-6)
Supplement: Additional file 1: Table S1. — Odds ratios for NAFLD in different quartiles of TyG index or ALT in ALT <40 and ≥40 U/L groups. Table S2 Diagnostic value of TyG and ALT for NAFLD in ALT <40 and ≥40 U/L groups. (DOCX 19 kb) [file 12944_2017_409_MOESM1_ESM.docx]

**Supplementary Table 1** Odds ratios for NAFLD in different quartiles of TyG index or ALT in ALT <40 and ≥40 U/L groups

| ALT <40 U/L |  |  |  |
| --- | --- | --- | --- |
|  |  | Unadjusted | Adjusted* |
| TyG | Q1 | 1 | 1 |
|  | Q2 (OR, 95% CI) | 2.9 (2.5-3.4) | 1.7 (1.4-2.1) |
|  | Q3 (OR, 95% CI) | 6.2 (5.3-7.3) | 2.7 (2.3-3.3) |
|  | Q4 (OR, 95% CI) | 17.1 (14.6-20.1) | 5.6 (4.7-6.8) |
| ALT | Q1 | 1 | 1 |
|  | Q2 (OR, 95% CI) | 1.9 (1.7-2.2) | 1.3 (1.1-1.5) |
|  | Q3 (OR, 95% CI) | 3.4 (3.0-3.9) | 1.8 (1.5-2.2) |
|  | Q4 (OR, 95% CI) | 6.1 (5.3-7.0) | 2.4 (2.0-2.9) |
| ALT ≥40 U/L |  |  |  |
|  |  | Unadjusted | Adjusted* |
| TyG | Q1 | 1 | 1 |
|  | Q2 (OR, 95% CI) | 4.4 (3.2-6.1) | 2.4 (1.6-3.7) |
|  | Q3 (OR, 95% CI) | 8.3 (5.8-11.9) | 3.8 (2.5-6.0) |
|  | Q4 (OR, 95% CI) | 17.0 (11.0-26.4) | 7.3 (4.3-12.3) |
| ALT | Q1 | 1 | 1 |
|  | Q2 (OR, 95% CI) | 1.3 (1.0-1.8) | 1.2 (0.8-1.9) |
|  | Q3 (OR, 95% CI) | 1.7 (1.2-2.4) | 1.5 (0.9-2.3) |
|  | Q4 (OR, 95% CI) | 1.7 (1.2-2.4) | 1.2 (0.7-1.8) |

*Adjusted for age, sex, body mass index, systolic blood pressure, uric acid, white blood cell counts, and ALT for TyG quartiles or TyG for ALT quartiles.

NAFLD, nonalcoholic fatty liver disease; ALT, alanine aminotransferase; Q1, first quartile; Q2, second quartile; Q3, third quartile; Q4, fourth quartile

**Supplementary Table 2** Diagnostic value of TyG and ALT for NAFLD in ALT <40 and ≥40 U/L groups

|  | AUC | | | 95%CI | Cut-off point | Sensitivity (%) | Specificity (%) |
| --- | --- | --- | --- | --- | --- | --- | --- |
| ALT <40 U/L | | |  |  |  |  |  |
| TyG | 0.769 | | | 0.759-0.779 | 8.5 | 67.8 | 73.0 |
| ALT | 0.684 | | | 0.673-0.696 | 18.5 | 66.4 | 61.3 |
| ALT ≥40U/L | |  | |  |  |  |  |
| TyG | 0.787 | | | 0.760-0.815 | 8.5 | 81.4 | 64.6 |
| ALT | 0.562 | | | 0.527-0.596 | 51.5 | 54.3 | 56.8 |

NAFLD, nonalcoholic fatty liver disease; ALT, alanine aminotransferase; AUC, areas under the curve
